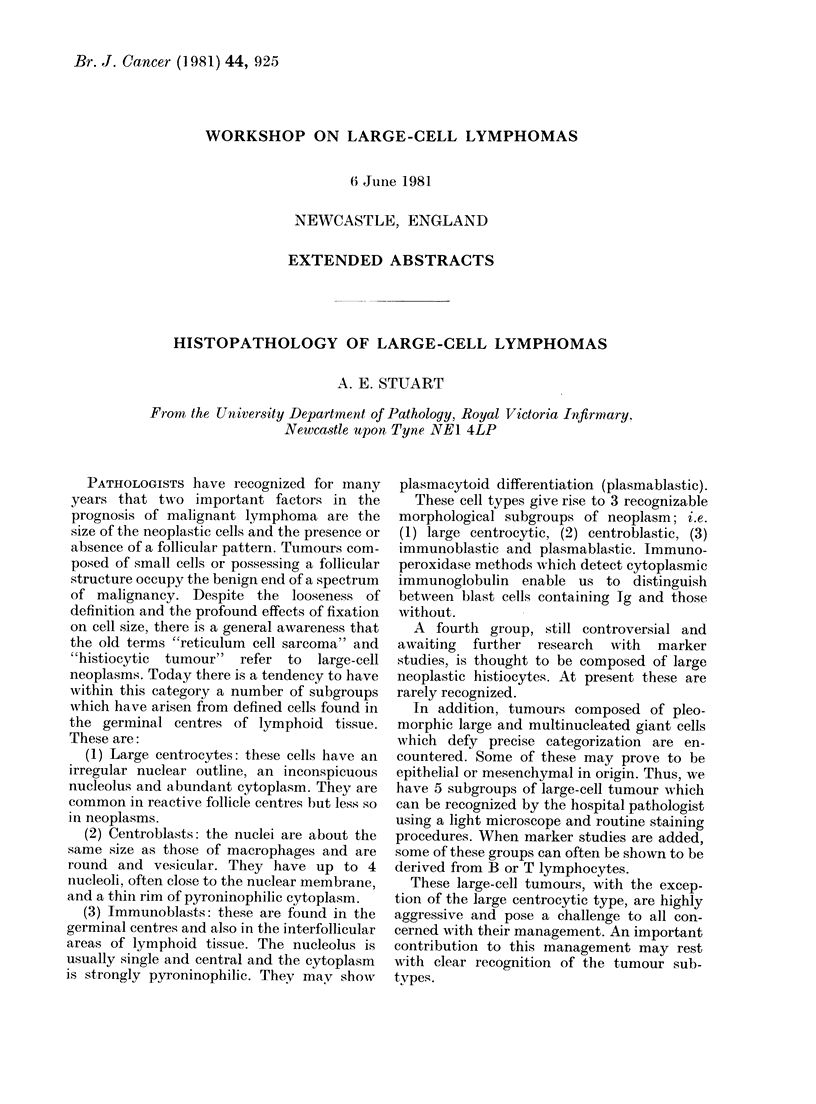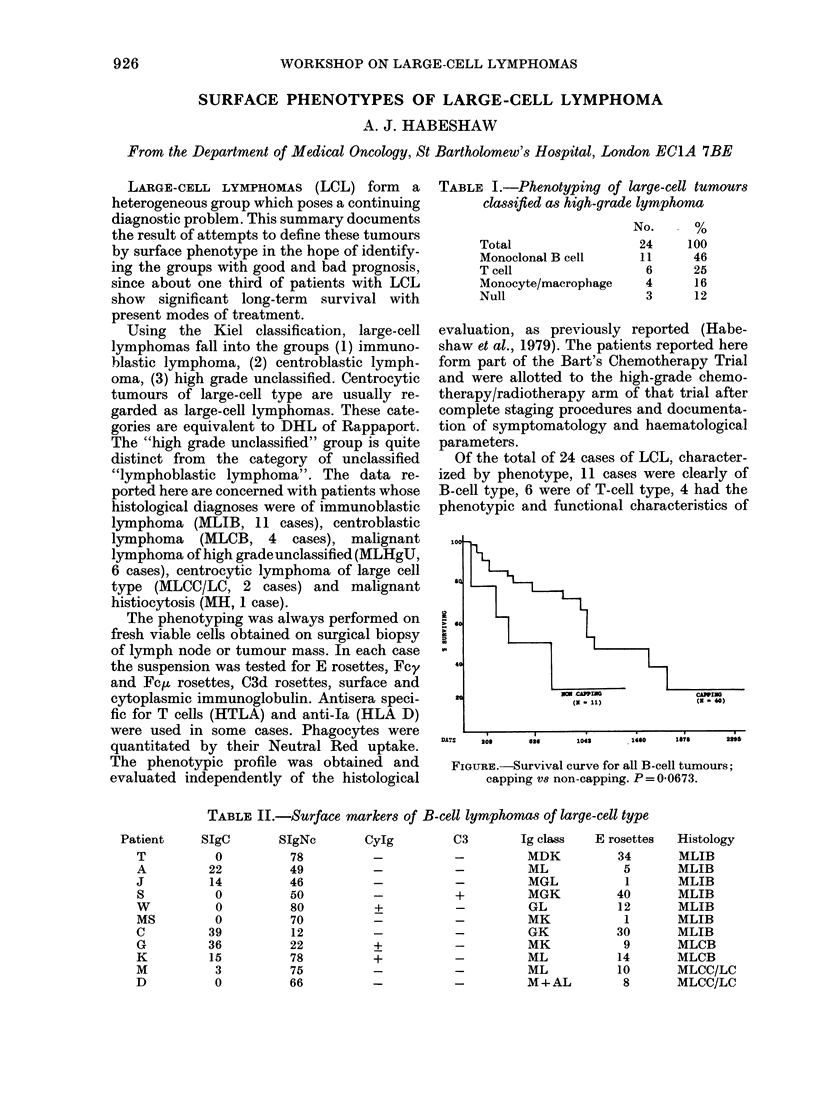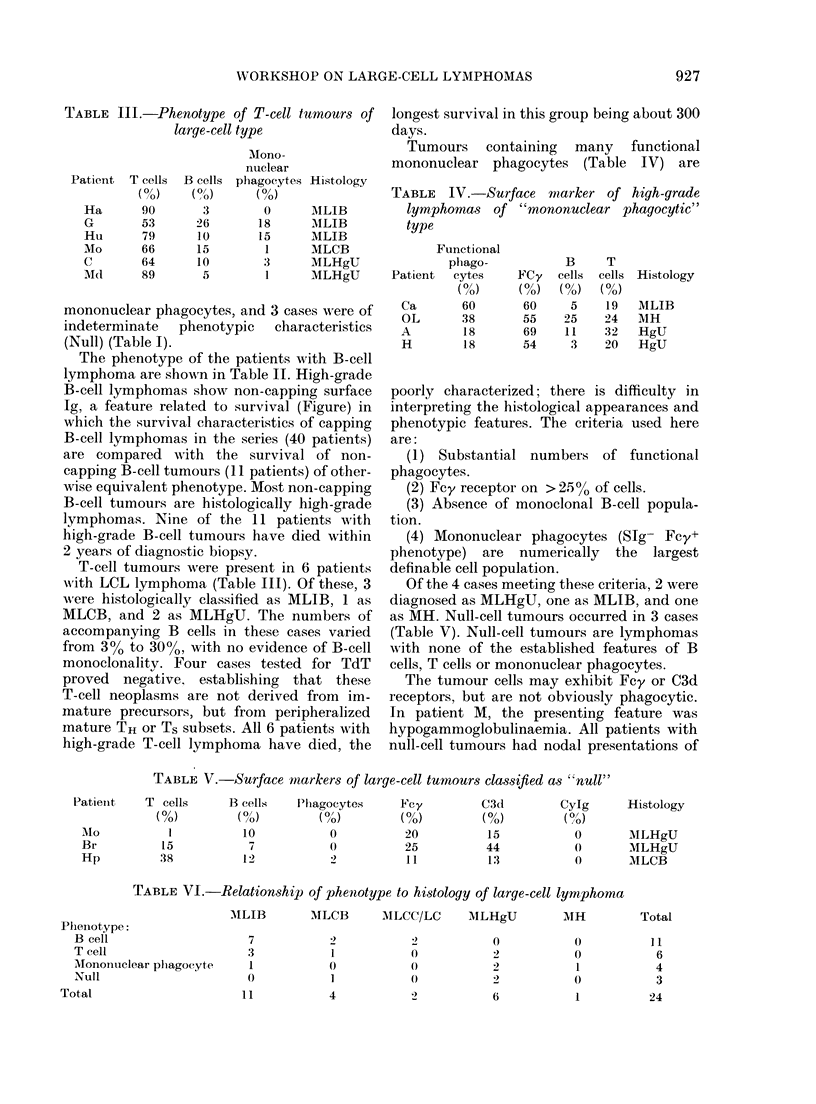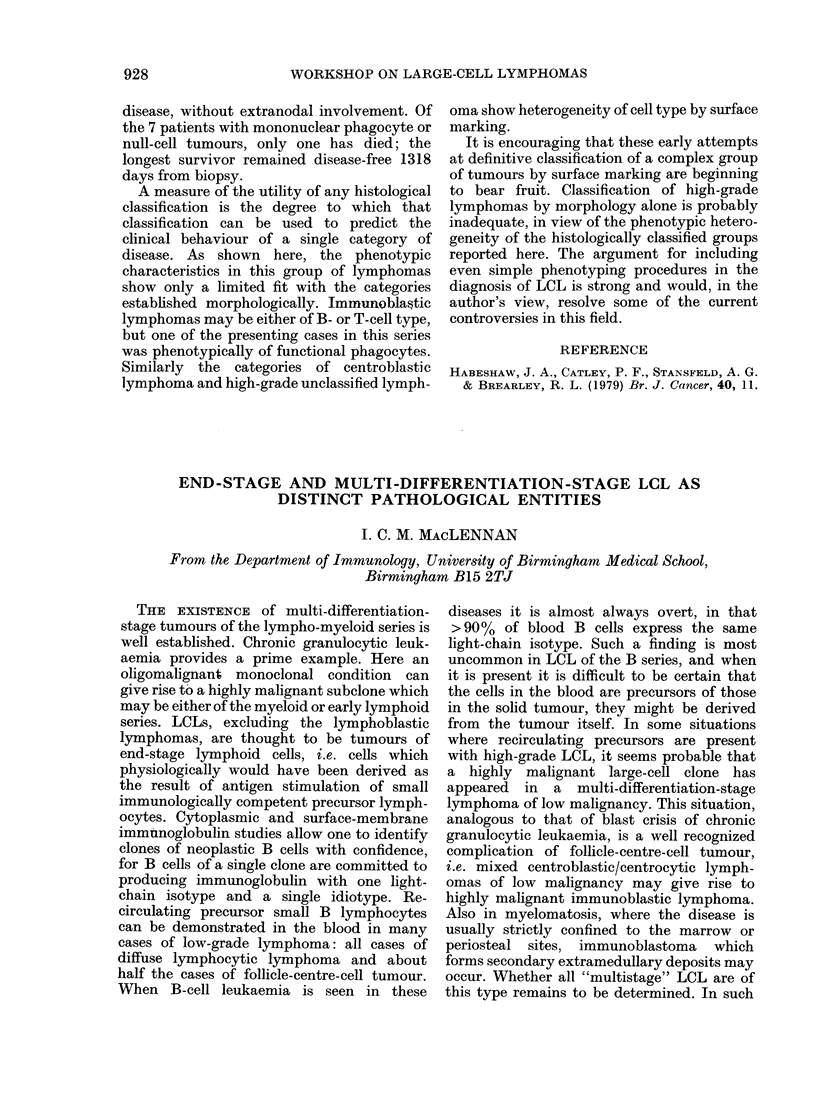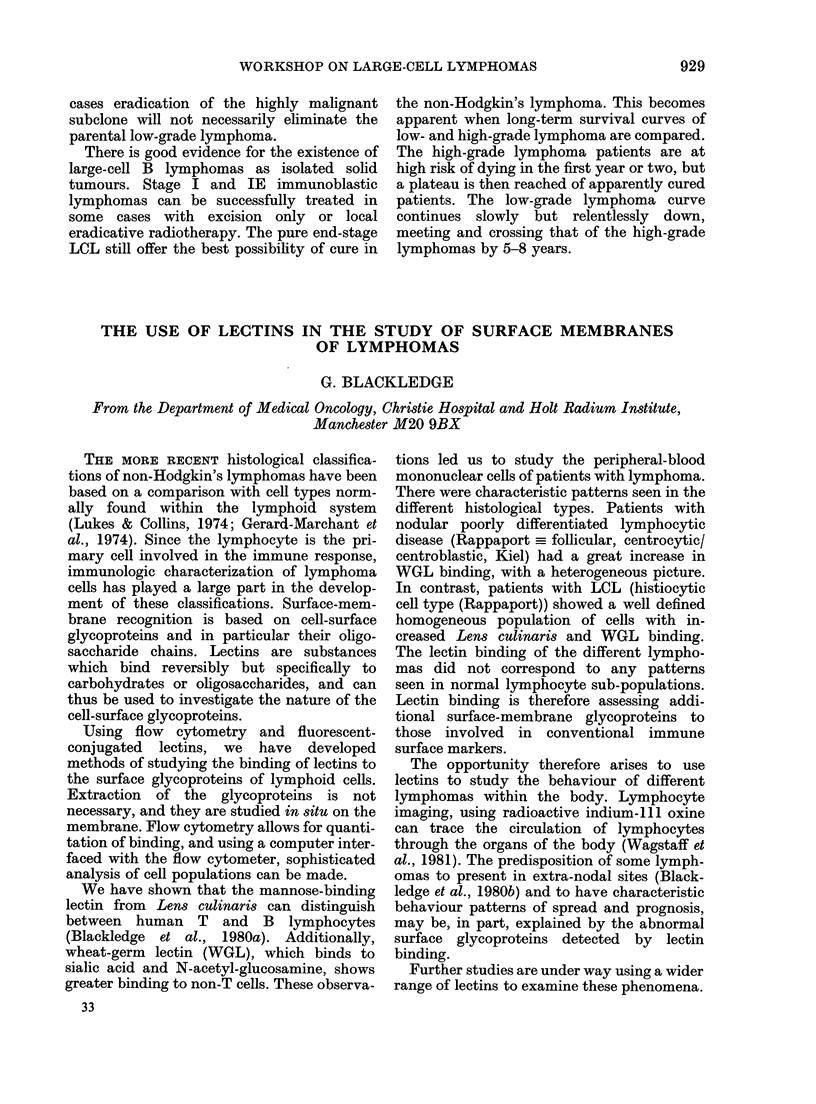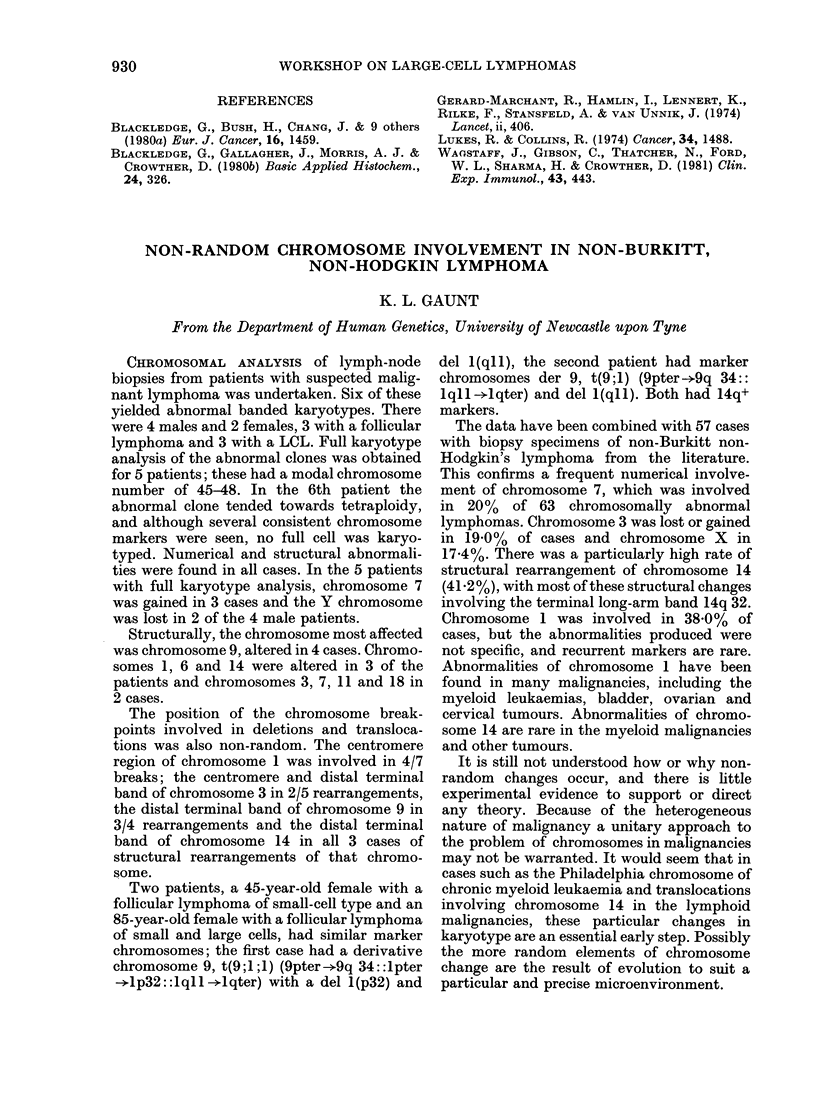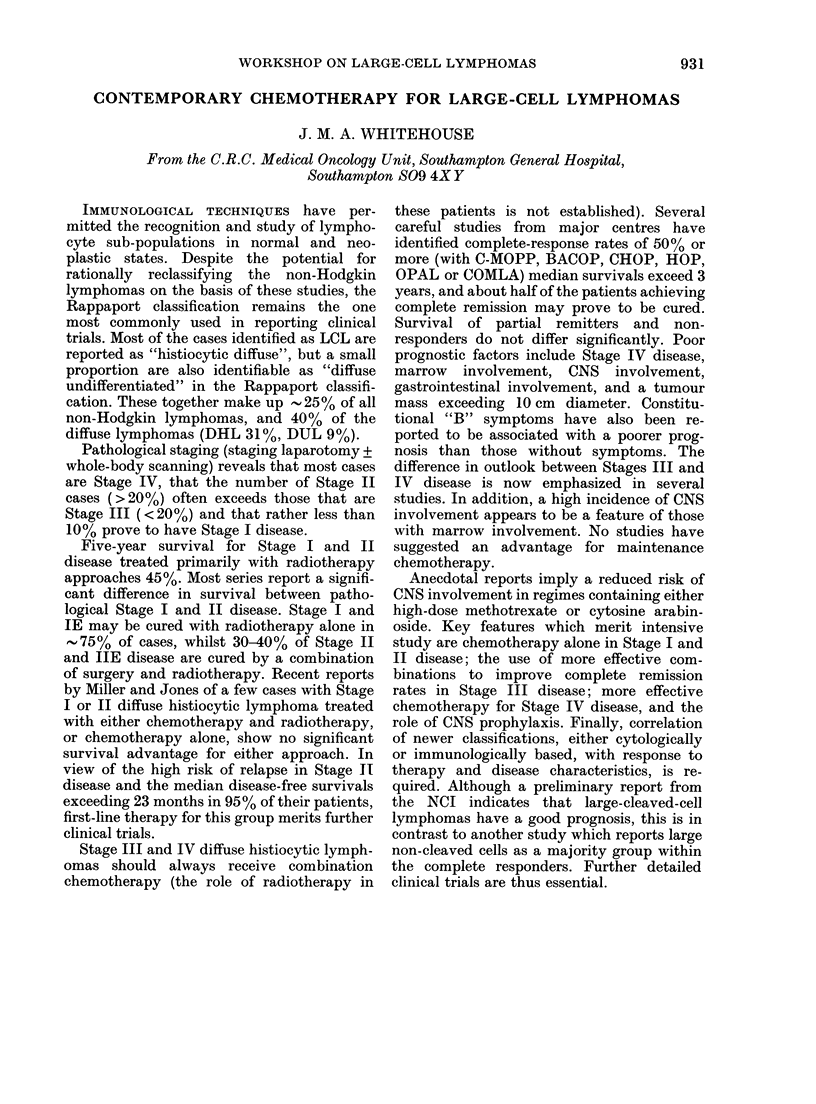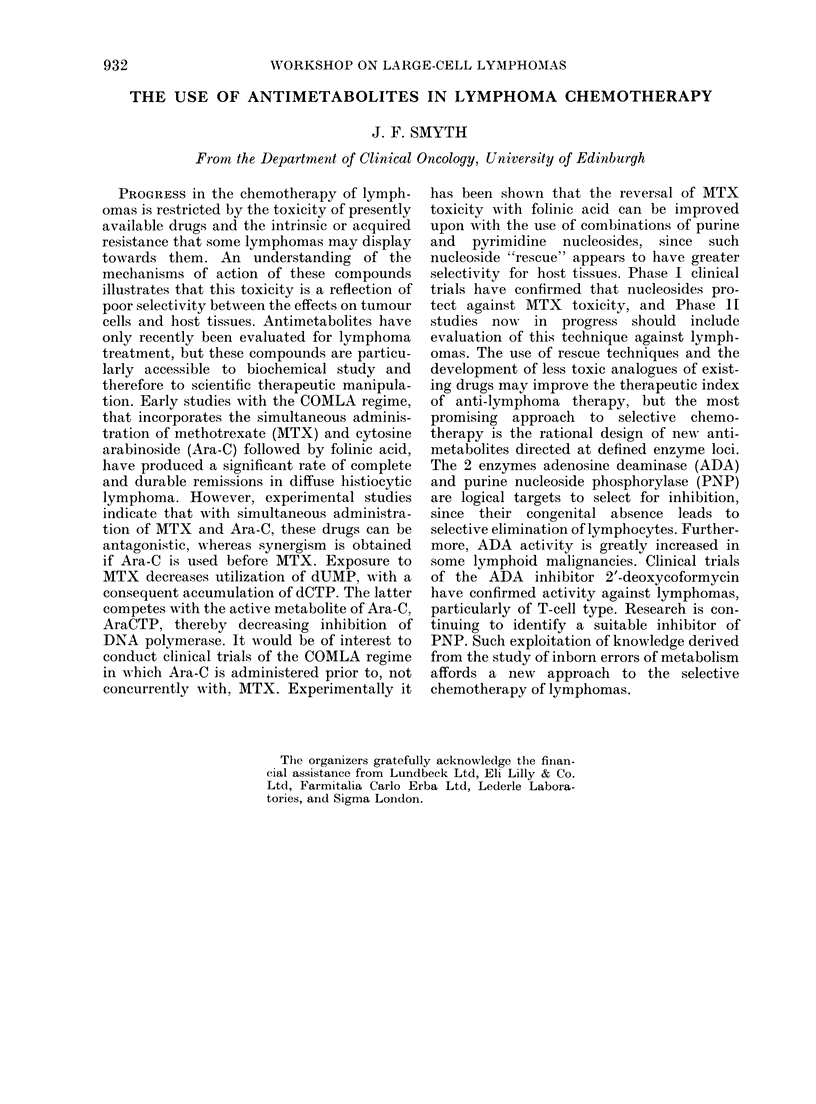# Workshop on large-cell lymphomas. 6 June 1981, Newcastle, England. Extended abstracts.

**DOI:** 10.1038/bjc.1981.294

**Published:** 1981-12

**Authors:** 


*Br. J. Cancer* (1981) **44**, 925

### WORKSHOP ON LARGE-CELL LYMPHOMAS

6 June 1981

NEWCASTLE, ENGLAND

### EXTENDED ABSTRACTS

---

### HISTOPATHOLOGY OF LARGE-CELL LYMPHOMAS

A. E. STUART

*From the University Department of Pathology, Royal Victoria Infirmary, Newcastle upon Tyne NE1 4LP*

PATHOLOGISTS have recognized for many years that two important factors in the prognosis of malignant lymphoma are the size of the neoplastic cells and the presence or absence of a follicular pattern. Tumours composed of small cells or possessing a follicular structure occupy the benign end of a spectrum of malignancy. Despite the looseness of definition and the profound effects of fixation on cell size, there is a general awareness that the old terms "reticulum cell sarcoma" and "histiocytic tumour" refer to large-cell neoplasms. Today there is a tendency to have within this category a number of subgroups which have arisen from defined cells found in the germinal centres of lymphoid tissue. These are:

(1) Large centrocytes: these cells have an irregular nuclear outline, an inconspicuous nucleolus and abundant cytoplasm. They are common in reactive follicle centres but less so in neoplasms.

(2) Centroblasts: the nuclei are about the same size as those of macrophages and are round and vesicular. They have up to 4 nucleoli, often close to the nuclear membrane, and a thin rim of pyroninophilic cytoplasm.

(3) Immunoblasts: these are found in the germinal centres and also in the interfollicular areas of lymphoid tissue. The nucleolus is usually single and central and the cytoplasm is strongly pyroninophilic. They may show plasmacytoid differentiation (plasmablastic).

These cell types give rise to 3 recognizable morphological subgroups of neoplasm; *i.e.* (1) large centrocytic, (2) centroblastic, (3) immunoblastic and plasmablastic. Immunoperoxidase methods which detect cytoplasmic immunoglobulin enable us to distinguish between blast cells containing Ig and those without.

A fourth group, still controversial and awaiting further research with marker studies, is thought to be composed of large neoplastic histiocytes. At present these are rarely recognized.

In addition, tumours composed of pleomorphic large and multinucleated giant cells which defy precise categorization are encountered. Some of these may prove to be epithelial or mesenchymal in origin. Thus, we have 5 subgroups of large-cell tumour which can be recognized by the hospital pathologist using a light microscope and routine staining procedures. When marker studies are added, some of these groups can often be shown to be derived from B or T lymphocytes.

These large-cell tumours, with the exception of the large centrocytic type, are highly aggressive and pose a challenge to all concerned with their management. An important contribution to this management may rest with clear recognition of the tumour subtypes.

### SURFACE PHENOTYPES OF LARGE-CELL LYMPHOMA

A. J. HABESHAW

*From the Department of Medical Oncology, St Bartholomew's Hospital, London EC1A 7BE*

LARGE-CELL LYMPHOMAS (LCL) form a heterogeneous group which poses a continuing diagnostic problem. This summary documents the result of attempts to define these tumours by surface phenotype in the hope of identifying the groups with good and bad prognosis, since about one third of patients with LCL show significant long-term survival with present modes of treatment.

Using the Kiel classification, large-cell lymphomas fall into the groups (1) immunoblastic lymphoma, (2) centroblastic lymphoma, (3) high grade unclassified. Centrocytic tumours of large-cell type are usually regarded as large-cell lymphomas. These categories are equivalent to DHL of Rappaport. The "high grade unclassified" group is quite distinct from the category of unclassified "lymphoblastic lymphoma". The data reported here are concerned with patients whose histological diagnoses were of immunoblastic lymphoma (MLIB, 11 cases), centroblastic lymphoma (MLCB, 4 cases), malignant lymphoma of high grade unclassified (MLHgU, 6 cases), centrocytic lymphoma of large cell type (MLCC/LC, 2 cases) and malignant histiocytosis (MH, 1 case).

The phenotyping was always performed on fresh viable cells obtained on surgical biopsy of lymph node or tumour mass. In each case the suspension was tested for E rosettes, Fc$\gamma$ and Fc$\mu$ rosettes, C3d rosettes, surface and cytoplasmic immunoglobulin. Antisera specific for T cells (HTLA) and anti-Ia (HLA D) were used in some cases. Phagocytes were quantitated by their Neutral Red uptake. The phenotypic profile was obtained and evaluated independently of the histological evaluation, as previously reported (Habeshaw *et al.*, 1979). The patients reported here form part of the Bart's Chemotherapy Trial and were allotted to the high-grade chemotherapy/radiotherapy arm of that trial after complete staging procedures and documentation of symptomatology and haematological parameters.

TABLE I.—*Phenotyping of large-cell tumours classified as high-grade lymphoma*

| | No. | % |
|---|---|---|
| Total | 24 | 100 |
| Monoclonal B cell | 11 | 46 |
| T cell | 6 | 25 |
| Monocyte/macrophage | 4 | 16 |
| Null | 3 | 12 |

Of the total of 24 cases of LCL, characterized by phenotype, 11 cases were clearly of B-cell type, 6 were of T-cell type, 4 had the phenotypic and functional characteristics of

FIGURE.—Survival curve for all B-cell tumours; capping *vs* non-capping. $P = 0{\cdot}0673$.

TABLE II.—*Surface markers of B-cell lymphomas of large-cell type*

| Patient | SIgC | SIgNc | CyIg | C3 | Ig class | E rosettes | Histology |
|---|---|---|---|---|---|---|---|
| T | 0 | 78 | – | – | MDK | 34 | MLIB |
| A | 22 | 49 | – | – | ML | 5 | MLIB |
| J | 14 | 46 | – | – | MGL | 1 | MLIB |
| S | 0 | 50 | – | + | MGK | 40 | MLIB |
| W | 0 | 80 | ± | – | GL | 12 | MLIB |
| MS | 0 | 70 | – | – | MK | 1 | MLIB |
| C | 39 | 12 | – | – | GK | 30 | MLIB |
| G | 36 | 22 | ± | – | MK | 9 | MLCB |
| K | 15 | 78 | + | – | ML | 14 | MLCB |
| M | 3 | 75 | – | – | ML | 10 | MLCC/LC |
| D | 0 | 66 | – | – | M+AL | 8 | MLCC/LC |

TABLE III.—*Phenotype of T-cell tumours of large-cell type*

| Patient | T cells (%) | B cells (%) | Mono-nuclear phagocytes (%) | Histology |
|---|---|---|---|---|
| Ha | 90 | 3 | 0 | MLIB |
| G | 53 | 26 | 18 | MLIB |
| Hu | 79 | 10 | 15 | MLIB |
| Mo | 66 | 15 | 1 | MLCB |
| C | 64 | 10 | 3 | MLHgU |
| Md | 89 | 5 | 1 | MLHgU |

mononuclear phagocytes, and 3 cases were of indeterminate phenotypic characteristics (Null) (Table I).

The phenotype of the patients with B-cell lymphoma are shown in Table II. High-grade B-cell lymphomas show non-capping surface Ig, a feature related to survival (Figure) in which the survival characteristics of capping B-cell lymphomas in the series (40 patients) are compared with the survival of non-capping B-cell tumours (11 patients) of otherwise equivalent phenotype. Most non-capping B-cell tumours are histologically high-grade lymphomas. Nine of the 11 patients with high-grade B-cell tumours have died within 2 years of diagnostic biopsy.

T-cell tumours were present in 6 patients with LCL lymphoma (Table III). Of these, 3 were histologically classified as MLIB, 1 as MLCB, and 2 as MLHgU. The numbers of accompanying B cells in these cases varied from 3% to 30%, with no evidence of B-cell monoclonality. Four cases tested for TdT proved negative, establishing that these T-cell neoplasms are not derived from immature precursors, but from peripheralized mature $T_H$ or $T_S$ subsets. All 6 patients with high-grade T-cell lymphoma have died, the longest survival in this group being about 300 days.

Tumours containing many functional mononuclear phagocytes (Table IV) are

TABLE IV.—*Surface marker of high-grade lymphomas of "mononuclear phagocytic" type*

| Patient | Functional phago-cytes (%) | FC$\gamma$ (%) | B cells (%) | T cells (%) | Histology |
|---|---|---|---|---|---|
| Ca | 60 | 60 | 5 | 19 | MLIB |
| OL | 38 | 55 | 25 | 24 | MH |
| A | 18 | 69 | 11 | 32 | HgU |
| H | 18 | 54 | 3 | 20 | HgU |

poorly characterized; there is difficulty in interpreting the histological appearances and phenotypic features. The criteria used here are:

(1) Substantial numbers of functional phagocytes.

(2) Fc$\gamma$ receptor on >25% of cells.

(3) Absence of monoclonal B-cell population.

(4) Mononuclear phagocytes ($SIg^-$ $Fc\gamma^+$ phenotype) are numerically the largest definable cell population.

Of the 4 cases meeting these criteria, 2 were diagnosed as MLHgU, one as MLIB, and one as MH. Null-cell tumours occurred in 3 cases (Table V). Null-cell tumours are lymphomas with none of the established features of B cells, T cells or mononuclear phagocytes.

The tumour cells may exhibit Fc$\gamma$ or C3d receptors, but are not obviously phagocytic. In patient M, the presenting feature was hypogammoglobulinaemia. All patients with null-cell tumours had nodal presentations of

TABLE V.—*Surface markers of large-cell tumours classified as "null"*

| Patient | T cells (%) | B cells (%) | Phagocytes (%) | Fc$\gamma$ (%) | C3d (%) | CyIg (%) | Histology |
|---|---|---|---|---|---|---|---|
| Mo | 1 | 10 | 0 | 20 | 15 | 0 | MLHgU |
| Br | 15 | 7 | 0 | 25 | 44 | 0 | MLHgU |
| Hp | 38 | 12 | 2 | 11 | 13 | 0 | MLCB |

TABLE VI.—*Relationship of phenotype to histology of large-cell lymphoma*

| | MLIB | MLCB | MLCC/LC | MLHgU | MH | Total |
|---|---|---|---|---|---|---|
| Phenotype: | | | | | | |
| B cell | 7 | 2 | 2 | 0 | 0 | 11 |
| T cell | 3 | 1 | 0 | 2 | 0 | 6 |
| Mononuclear phagocyte | 1 | 0 | 0 | 2 | 1 | 4 |
| Null | 0 | 1 | 0 | 2 | 0 | 3 |
| Total | 11 | 4 | 2 | 6 | 1 | 24 |

disease, without extranodal involvement. Of the 7 patients with mononuclear phagocyte or null-cell tumours, only one has died; the longest survivor remained disease-free 1318 days from biopsy.

A measure of the utility of any histological classification is the degree to which that classification can be used to predict the clinical behaviour of a single category of disease. As shown here, the phenotypic characteristics in this group of lymphomas show only a limited fit with the categories established morphologically. Immunoblastic lymphomas may be either of B- or T-cell type, but one of the presenting cases in this series was phenotypically of functional phagocytes. Similarly the categories of centroblastic lymphoma and high-grade unclassified lymphoma show heterogeneity of cell type by surface marking.

It is encouraging that these early attempts at definitive classification of a complex group of tumours by surface marking are beginning to bear fruit. Classification of high-grade lymphomas by morphology alone is probably inadequate, in view of the phenotypic heterogeneity of the histologically classified groups reported here. The argument for including even simple phenotyping procedures in the diagnosis of LCL is strong and would, in the author's view, resolve some of the current controversies in this field.

REFERENCE

HABESHAW, J. A., CATLEY, P. F., STANSFELD, A. G. & BREARLEY, R. L. (1979) *Br. J. Cancer*, **40**, 11.

### END-STAGE AND MULTI-DIFFERENTIATION-STAGE LCL AS DISTINCT PATHOLOGICAL ENTITIES

I. C. M. MACLENNAN

*From the Department of Immunology, University of Birmingham Medical School, Birmingham B15 2TJ*

THE EXISTENCE of multi-differentiation-stage tumours of the lympho-myeloid series is well established. Chronic granulocytic leukaemia provides a prime example. Here an oligomalignant monoclonal condition can give rise to a highly malignant subclone which may be either of the myeloid or early lymphoid series. LCLs, excluding the lymphoblastic lymphomas, are thought to be tumours of end-stage lymphoid cells, *i.e.* cells which physiologically would have been derived as the result of antigen stimulation of small immunologically competent precursor lymphocytes. Cytoplasmic and surface-membrane immunoglobulin studies allow one to identify clones of neoplastic B cells with confidence, for B cells of a single clone are committed to producing immunoglobulin with one light-chain isotype and a single idiotype. Recirculating precursor small B lymphocytes can be demonstrated in the blood in many cases of low-grade lymphoma: all cases of diffuse lymphocytic lymphoma and about half the cases of follicle-centre-cell tumour. When B-cell leukaemia is seen in these diseases it is almost always overt, in that $>90\%$ of blood B cells express the same light-chain isotype. Such a finding is most uncommon in LCL of the B series, and when it is present it is difficult to be certain that the cells in the blood are precursors of those in the solid tumour, they might be derived from the tumour itself. In some situations where recirculating precursors are present with high-grade LCL, it seems probable that a highly malignant large-cell clone has appeared in a multi-differentiation-stage lymphoma of low malignancy. This situation, analogous to that of blast crisis of chronic granulocytic leukaemia, is a well recognized complication of follicle-centre-cell tumour, *i.e.* mixed centroblastic/centrocytic lymphomas of low malignancy may give rise to highly malignant immunoblastic lymphoma. Also in myelomatosis, where the disease is usually strictly confined to the marrow or periosteal sites, immunoblastoma which forms secondary extramedullary deposits may occur. Whether all "multistage" LCL are of this type remains to be determined. In such cases eradication of the highly malignant subclone will not necessarily eliminate the parental low-grade lymphoma.

There is good evidence for the existence of large-cell B lymphomas as isolated solid tumours. Stage I and IE immunoblastic lymphomas can be successfully treated in some cases with excision only or local eradicative radiotherapy. The pure end-stage LCL still offer the best possibility of cure in the non-Hodgkin's lymphoma. This becomes apparent when long-term survival curves of low- and high-grade lymphoma are compared. The high-grade lymphoma patients are at high risk of dying in the first year or two, but a plateau is then reached of apparently cured patients. The low-grade lymphoma curve continues slowly but relentlessly down, meeting and crossing that of the high-grade lymphomas by 5–8 years.

### THE USE OF LECTINS IN THE STUDY OF SURFACE MEMBRANES OF LYMPHOMAS

G. BLACKLEDGE

*From the Department of Medical Oncology, Christie Hospital and Holt Radium Institute, Manchester M20 9BX*

THE MORE RECENT histological classifications of non-Hodgkin's lymphomas have been based on a comparison with cell types normally found within the lymphoid system (Lukes & Collins, 1974; Gerard-Marchant *et al.*, 1974). Since the lymphocyte is the primary cell involved in the immune response, immunologic characterization of lymphoma cells has played a large part in the development of these classifications. Surface-membrane recognition is based on cell-surface glycoproteins and in particular their oligosaccharide chains. Lectins are substances which bind reversibly but specifically to carbohydrates or oligosaccharides, and can thus be used to investigate the nature of the cell-surface glycoproteins.

Using flow cytometry and fluorescent-conjugated lectins, we have developed methods of studying the binding of lectins to the surface glycoproteins of lymphoid cells. Extraction of the glycoproteins is not necessary, and they are studied *in situ* on the membrane. Flow cytometry allows for quantitation of binding, and using a computer interfaced with the flow cytometer, sophisticated analysis of cell populations can be made.

We have shown that the mannose-binding lectin from *Lens culinaris* can distinguish between human T and B lymphocytes (Blackledge *et al.*, 1980*a*). Additionally, wheat-germ lectin (WGL), which binds to sialic acid and N-acetyl-glucosamine, shows greater binding to non-T cells. These observations led us to study the peripheral-blood mononuclear cells of patients with lymphoma. There were characteristic patterns seen in the different histological types. Patients with nodular poorly differentiated lymphocytic disease (Rappaport ≡ follicular, centrocytic/centroblastic, Kiel) had a great increase in WGL binding, with a heterogeneous picture. In contrast, patients with LCL (histiocytic cell type (Rappaport)) showed a well defined homogeneous population of cells with increased *Lens culinaris* and WGL binding. The lectin binding of the different lymphomas did not correspond to any patterns seen in normal lymphocyte sub-populations. Lectin binding is therefore assessing additional surface-membrane glycoproteins to those involved in conventional immune surface markers.

The opportunity therefore arises to use lectins to study the behaviour of different lymphomas within the body. Lymphocyte imaging, using radioactive indium-111 oxine can trace the circulation of lymphocytes through the organs of the body (Wagstaff *et al.*, 1981). The predisposition of some lymphomas to present in extra-nodal sites (Blackledge *et al.*, 1980*b*) and to have characteristic behaviour patterns of spread and prognosis, may be, in part, explained by the abnormal surface glycoproteins detected by lectin binding.

Further studies are under way using a wider range of lectins to examine these phenomena.

REFERENCES

BLACKLEDGE, G., BUSH, H., CHANG, J. & 9 others (1980*a*) *Eur. J. Cancer*, **16**, 1459.

BLACKLEDGE, G., GALLAGHER, J., MORRIS, A. J. & CROWTHER, D. (1980*b*) *Basic Applied Histochem.*, **24**, 326.

GERARD-MARCHANT, R., HAMLIN, I., LENNERT, K., RILKE, F., STANSFELD, A. & VAN UNNIK, J. (1974) *Lancet*, ii, 406.

LUKES, R. & COLLINS, R. (1974) *Cancer*, **34**, 1488.

WAGSTAFF, J., GIBSON, C., THATCHER, N., FORD, W. L., SHARMA, H. & CROWTHER, D. (1981) *Clin. Exp. Immunol.*, **43**, 443.

### NON-RANDOM CHROMOSOME INVOLVEMENT IN NON-BURKITT, NON-HODGKIN LYMPHOMA

K. L. GAUNT

*From the Department of Human Genetics, University of Newcastle upon Tyne*

CHROMOSOMAL ANALYSIS of lymph-node biopsies from patients with suspected malignant lymphoma was undertaken. Six of these yielded abnormal banded karyotypes. There were 4 males and 2 females, 3 with a follicular lymphoma and 3 with a LCL. Full karyotype analysis of the abnormal clones was obtained for 5 patients; these had a modal chromosome number of 45–48. In the 6th patient the abnormal clone tended towards tetraploidy, and although several consistent chromosome markers were seen, no full cell was karyotyped. Numerical and structural abnormalities were found in all cases. In the 5 patients with full karyotype analysis, chromosome 7 was gained in 3 cases and the Y chromosome was lost in 2 of the 4 male patients.

Structurally, the chromosome most affected was chromosome 9, altered in 4 cases. Chromosomes 1, 6 and 14 were altered in 3 of the patients and chromosomes 3, 7, 11 and 18 in 2 cases.

The position of the chromosome breakpoints involved in deletions and translocations was also non-random. The centromere region of chromosome 1 was involved in 4/7 breaks; the centromere and distal terminal band of chromosome 3 in 2/5 rearrangements, the distal terminal band of chromosome 9 in 3/4 rearrangements and the distal terminal band of chromosome 14 in all 3 cases of structural rearrangements of that chromosome.

Two patients, a 45-year-old female with a follicular lymphoma of small-cell type and an 85-year-old female with a follicular lymphoma of small and large cells, had similar marker chromosomes; the first case had a derivative chromosome 9, t(9;1;1) (9pter→9q 34::1pter→1p32::1q11→1qter) with a del 1(p32) and del 1(q11), the second patient had marker chromosomes der 9, t(9;1) (9pter→9q 34::1q11→1qter) and del 1(q11). Both had $14q^+$ markers.

The data have been combined with 57 cases with biopsy specimens of non-Burkitt non-Hodgkin's lymphoma from the literature. This confirms a frequent numerical involvement of chromosome 7, which was involved in 20% of 63 chromosomally abnormal lymphomas. Chromosome 3 was lost or gained in 19·0% of cases and chromosome X in 17·4%. There was a particularly high rate of structural rearrangement of chromosome 14 (41·2%), with most of these structural changes involving the terminal long-arm band 14q 32. Chromosome 1 was involved in 38·0% of cases, but the abnormalities produced were not specific, and recurrent markers are rare. Abnormalities of chromosome 1 have been found in many malignancies, including the myeloid leukaemias, bladder, ovarian and cervical tumours. Abnormalities of chromosome 14 are rare in the myeloid malignancies and other tumours.

It is still not understood how or why non-random changes occur, and there is little experimental evidence to support or direct any theory. Because of the heterogeneous nature of malignancy a unitary approach to the problem of chromosomes in malignancies may not be warranted. It would seem that in cases such as the Philadelphia chromosome of chronic myeloid leukaemia and translocations involving chromosome 14 in the lymphoid malignancies, these particular changes in karyotype are an essential early step. Possibly the more random elements of chromosome change are the result of evolution to suit a particular and precise microenvironment.

### CONTEMPORARY CHEMOTHERAPY FOR LARGE-CELL LYMPHOMAS

J. M. A. WHITEHOUSE

*From the C.R.C. Medical Oncology Unit, Southampton General Hospital, Southampton SO9 4XY*

IMMUNOLOGICAL TECHNIQUES have permitted the recognition and study of lymphocyte sub-populations in normal and neoplastic states. Despite the potential for rationally reclassifying the non-Hodgkin lymphomas on the basis of these studies, the Rappaport classification remains the one most commonly used in reporting clinical trials. Most of the cases identified as LCL are reported as "histiocytic diffuse", but a small proportion are also identifiable as "diffuse undifferentiated" in the Rappaport classification. These together make up ~25% of all non-Hodgkin lymphomas, and 40% of the diffuse lymphomas (DHL 31%, DUL 9%).

Pathological staging (staging laparotomy ± whole-body scanning) reveals that most cases are Stage IV, that the number of Stage II cases (>20%) often exceeds those that are Stage III (<20%) and that rather less than 10% prove to have Stage I disease.

Five-year survival for Stage I and II disease treated primarily with radiotherapy approaches 45%. Most series report a significant difference in survival between pathological Stage I and II disease. Stage I and IE may be cured with radiotherapy alone in ~75% of cases, whilst 30–40% of Stage II and IIE disease are cured by a combination of surgery and radiotherapy. Recent reports by Miller and Jones of a few cases with Stage I or II diffuse histiocytic lymphoma treated with either chemotherapy and radiotherapy, or chemotherapy alone, show no significant survival advantage for either approach. In view of the high risk of relapse in Stage II disease and the median disease-free survivals exceeding 23 months in 95% of their patients, first-line therapy for this group merits further clinical trials.

Stage III and IV diffuse histiocytic lymphomas should always receive combination chemotherapy (the role of radiotherapy in these patients is not established). Several careful studies from major centres have identified complete-response rates of 50% or more (with C-MOPP, BACOP, CHOP, HOP, OPAL or COMLA) median survivals exceed 3 years, and about half of the patients achieving complete remission may prove to be cured. Survival of partial remitters and non-responders do not differ significantly. Poor prognostic factors include Stage IV disease, marrow involvement, CNS involvement, gastrointestinal involvement, and a tumour mass exceeding 10 cm diameter. Constitutional "B" symptoms have also been reported to be associated with a poorer prognosis than those without symptoms. The difference in outlook between Stages III and IV disease is now emphasized in several studies. In addition, a high incidence of CNS involvement appears to be a feature of those with marrow involvement. No studies have suggested an advantage for maintenance chemotherapy.

Anecdotal reports imply a reduced risk of CNS involvement in regimes containing either high-dose methotrexate or cytosine arabinoside. Key features which merit intensive study are chemotherapy alone in Stage I and II disease; the use of more effective combinations to improve complete remission rates in Stage III disease; more effective chemotherapy for Stage IV disease, and the role of CNS prophylaxis. Finally, correlation of newer classifications, either cytologically or immunologically based, with response to therapy and disease characteristics, is required. Although a preliminary report from the NCI indicates that large-cleaved-cell lymphomas have a good prognosis, this is in contrast to another study which reports large non-cleaved cells as a majority group within the complete responders. Further detailed clinical trials are thus essential.

### THE USE OF ANTIMETABOLITES IN LYMPHOMA CHEMOTHERAPY

J. F. SMYTH

*From the Department of Clinical Oncology, University of Edinburgh*

Progress in the chemotherapy of lymphomas is restricted by the toxicity of presently available drugs and the intrinsic or acquired resistance that some lymphomas may display towards them. An understanding of the mechanisms of action of these compounds illustrates that this toxicity is a reflection of poor selectivity between the effects on tumour cells and host tissues. Antimetabolites have only recently been evaluated for lymphoma treatment, but these compounds are particularly accessible to biochemical study and therefore to scientific therapeutic manipulation. Early studies with the COMLA regime, that incorporates the simultaneous administration of methotrexate (MTX) and cytosine arabinoside (Ara-C) followed by folinic acid, have produced a significant rate of complete and durable remissions in diffuse histiocytic lymphoma. However, experimental studies indicate that with simultaneous administration of MTX and Ara-C, these drugs can be antagonistic, whereas synergism is obtained if Ara-C is used before MTX. Exposure to MTX decreases utilization of dUMP, with a consequent accumulation of dCTP. The latter competes with the active metabolite of Ara-C, AraCTP, thereby decreasing inhibition of DNA polymerase. It would be of interest to conduct clinical trials of the COMLA regime in which Ara-C is administered prior to, not concurrently with, MTX. Experimentally it has been shown that the reversal of MTX toxicity with folinic acid can be improved upon with the use of combinations of purine and pyrimidine nucleosides, since such nucleoside "rescue" appears to have greater selectivity for host tissues. Phase I clinical trials have confirmed that nucleosides protect against MTX toxicity, and Phase II studies now in progress should include evaluation of this technique against lymphomas. The use of rescue techniques and the development of less toxic analogues of existing drugs may improve the therapeutic index of anti-lymphoma therapy, but the most promising approach to selective chemotherapy is the rational design of new antimetabolites directed at defined enzyme loci. The 2 enzymes adenosine deaminase (ADA) and purine nucleoside phosphorylase (PNP) are logical targets to select for inhibition, since their congenital absence leads to selective elimination of lymphocytes. Furthermore, ADA activity is greatly increased in some lymphoid malignancies. Clinical trials of the ADA inhibitor 2′-deoxycoformycin have confirmed activity against lymphomas, particularly of T-cell type. Research is continuing to identify a suitable inhibitor of PNP. Such exploitation of knowledge derived from the study of inborn errors of metabolism affords a new approach to the selective chemotherapy of lymphomas.

The organizers gratefully acknowledge the financial assistance from Lundbeck Ltd, Eli Lilly & Co. Ltd, Farmitalia Carlo Erba Ltd, Lederle Laboratories, and Sigma London.